# Changes in nutritional quality-related traits of quinoa seeds under different storage conditions

**DOI:** 10.3389/fnut.2022.995250

**Published:** 2022-10-17

**Authors:** Sara Granado-Rodríguez, Isaac Maestro-Gaitán, Javier Matías, María José Rodríguez, Patricia Calvo, Luis Eduardo Hernández, Luis Bolaños, Maria Reguera

**Affiliations:** ^1^Departamento de Biología, Universidad Autónoma de Madrid, Madrid, Spain; ^2^Centro de Investigaciones Científicas y Tecnológicas de Extremadura (CICYTEX), Agrarian Research Institute “La Orden-Valdesequera” of Extremadura, Guadajira, Spain; ^3^Technological Institute of Food and Agriculture of Extremadura, Centro de Investigaciones Científicas y Tecnológicas de Extremadura (CICYTEX), Guadajira, Spain

**Keywords:** quinoa seeds, nutritional traits, seed oil quality, post-harvest, seed germination, oxidative stress, shelf life

## Abstract

Within the context of climate change and its impact on global food security, seed storage has become key, as it ensures long-term food and next-season seed preservation. Aiming at evaluating quality-related changes in quinoa seeds over storage time, different storage temperatures (–20, 4, 12, 25, and 37°C) and humidity conditions (use of silica gel or not) were studied and different seed nutritional parameters were evaluated at different points during a year of storage. Also, to determine if these variations could be conditioned by the genotype used, two quinoa cultivars were compared. The results proved that quinoa seed quality is highly dependent on the storage temperature but is not consistently affected by the use of silica gel if the seed moisture content (SMC) is kept between 5 and 12%. Furthermore, quality can be maintained and even improved by keeping SMC lower than 12% and storage temperatures low (4°C). Under these conditions (at 4°C in hermetic packaging with or without silica gel), and after 12 months of storage, there was an increase in amino acids like isoleucine, serine, arginine, glycine, and glutamic acid and in seed viability and germination. On the contrary, quinoa seeds stored at 37°C showed an accumulation of reactive oxygen species (ROS) which was related to a lower antioxidant capacity and a reduction in the contents of essential amino acids like isoleucine, lysine, histidine, and threonine, resulting in a delayed and reduced germination capacity, and, therefore, lower seed quality. Besides, quality-related differences appeared between cultivars highlighting differences linked to the genotype. Overall, this work demonstrates that optimal storage temperatures and SMC can preserve or even improve quinoa seed nutritional quality, which in turn can impact food safety and agriculture.

## Introduction

The storage of foods is fundamental to ensure safe transportation to their final destination and, in the case of annual crops, to make their consumption outside of the harvest season possible. Foods consisting of seeds (including cereal grains or pseudocereals and legumes seeds) or their derivates have a great storability potential since they need to be preserved until their use in the next sowing season. They can be stored for longer periods and at lower costs compared to other food products like meat, dairy, or vegetables. The storage of seeds is key in the context of global food security and has been used both at a household and government level to guarantee access to food during trying times (1).

Adequate preservation of the food produced is indispensable for maintaining sustainable agriculture and avoiding economical losses, especially in the case of small farmers, due to the risks associated with post-harvest damage (2). This is particularly relevant in the case of small producers in developing countries since the infrastructure for storage and transportation is inadequate and is expected to worsen, particularly in these areas, as a consequence of climate change (3, 4).

Storage conditions are critical for ensuring the longevity of grains or seeds, specifically storage temperature, relative humidity (RH), and storage method (such as the use of bags or silos or the nature of the packaging material used), which directly affect seed moisture contents (SMC), and, to a lesser degree, the presence of oxygen (5, 6). Generally, lower SMC (ranging between 5 and 14% for most seeds, including cereals, pseudocereals, and pulses) and oxygen levels are required during seed storage to prevent the activation of cellular respiration and other metabolic processes such as reserve mobilization, which are potentially harmful to seed preservation (7, 8) affecting as well the proliferation of mold and other storage pests (9–11). High storage temperature is also a major contributor to seed aging, and can affect biochemical processes rates facilitating mold proliferation (6, 10). Furthermore, the biochemical changes that induce seed aging can also affect nutritional quality-related traits or even impact organoleptic characteristics (taste, smell, color) (12–14).

Most postharvest studies have been performed on cereals, since cereals such as wheat, maize, or rice are staple crops in many countries and account for, approximately, 85% of the global food production (15). However, the need for food sources diversification has been exposed for years now (16, 17), and recent events have exacerbated this need. For instance, wheat production has been gravely affected in the last years in different parts of the world as a consequence of extreme climatic events, like drought in Morocco or extreme heat waves in India and Pakistan (18, 19). This, together with the difficulties suffered in the food supply chain due to the sanitary crisis caused by the covid-19 pandemic, and the wheat export blockage linked to international conflicts (Russia and Ukraine account for 16.5 and 9.5% of the world wheat exportations, respectively), have caused a significant reduction in the access to food in wheat importer countries, especially in the Middle East, North of Africa and the Sahel (19, 20). This has highlighted, on one hand, the need of diversifying food sources to implement sustainable, equitable, and resilient food systems in which alternative crops may play fundamental roles (21), and on the other, the necessity of enabling longer food storage periods, keeping quality, thus contributing to increasing food security worldwide (22, 23).

Quinoa (*Chenopodium quinoa* Wild.) is a dicotyledonous crop from the Amaranthaceae family. It is considered a pseudocereal due to the similar food uses and starch-based composition of its seeds equivalent to those of cereal grains from the Poaceae family (24). Quinoa has gained huge popularity over the last few decades because of the excellent nutritional quality of its seeds (25, 26), which includes a high protein content with a balanced amino acid profile (27, 28), fats of good quality consisting mainly of polyunsaturated fatty acids (PUFAs) (29, 30), high contents of minerals, and compounds like polyphenols and flavonoids with antioxidant activity (31). Furthermore, quinoa is a resilient crop that can tolerate adverse growing conditions such as drought, saline, and low fertility soils (32). Thus, quinoa is a promising alternate crop for developing countries with marginal lands, facing harsh climates, and with few resources for ensuring adequate crop management (4, 33, 34), having the potential to contribute to achieving global food security (35). Currently, the cultivation of quinoa has expanded from its center of origin, located in the Andean Altiplano, to more than one hundred countries (35, 36). Still, Peru and Bolivia are the main quinoa global producers from where seeds are distributed all over the world, especially to Europe, Canada, and the US (37, 38). In this sense, the preservation of seed quality is key not only for quinoa farmers to ensure next season’s planting but also for quinoa consumers as the nutritional properties might be affected during storage.

Quinoa seed germination is affected by the humidity and temperature conditions during storage (39, 40). In fact, these two factors are determinants of the preservation of quinoa seeds as they not only affect the germinative power of seeds but also their dormancy and sprouting (41, 42). Furthermore, recently, it was shown that quinoa seed quality preservation (in terms of vigor, viability, lipid peroxidation and sugar content) is reduced when kept in traditional package materials or at moisture contents higher than 10% in hermetic bags due to biochemical changes, stressing out the possibility of a relation between the storage conditions and the nutritional quality (8). Nevertheless, how exactly the storage conditions affect the nutritional properties of quinoa seeds remains largely unknown and should be further explored.

Hence, aiming at providing further knowledge related to the changes in quality of quinoa seeds over storage time while considering the storage temperature and humidity factors, we analyzed the effect of different storage temperatures and humidity conditions on germination, seed viability, and several nutritional parameters (including the fatty acid profile, amino acids or antioxidants) to elucidate the impact of these factors on quinoa seed nutritional quality preservation. Furthermore, to analyze the possible contribution of the genotypic factor to the storage effects, seeds harvested from two quinoa cultivars were used in this study.

## Materials and methods

### Experimental design

A random design was used in a 2 × 2 × 5 factorial arrangement (two genotypes, two humidity conditions, and five temperatures). The seeds used came from two different *C. quinoa* cultivars, F16 and Duquesa, provided by Algosur S. L., (both cultivars grown under the same field environmental conditions in southern Spain latitudes) and harvested just before starting the experiment. Approximately, 200 g of seeds of each cultivar were kept in zipper hermetic polyethylene bags at two different humidity conditions (with or without 10 g of silica gel), and at five different temperatures of storage (–20, 4, 12, 25, and 37°C) in different chambers with stable temperature and humidity conditions. RH in the chambers was kept constant at 100, 90, 75, 60, and 50%, respectively. The bags were stored under dark conditions for 12 months. The silica gel packages used to control humidity were replaced periodically, as needed (approximately every 2 weeks, based on the color change of silica gel from blue to pink).

To evaluate changes in the seed’s nutritional properties, seed quality analyses were performed before storage (to determine the initial quality of the seeds) and 3, 6, and 12 months after storage. For each measurement, at least three replicates were used per genotype, temperature, humidity, and time of storage.

### Seed moisture content

A hundred seeds were manually counted and weighed using an analytical balance before (fresh) and after drying them in an oven for 17 h at 103°C (to obtain the dry weight) (43). Seed moisture was then calculated following the equation $S\,M\,C=\frac{F\,r\,e\,s\,h\,w\,i\,e\,g\,h\,t-D\,r\,y\,w\,e\,i\,g\,h\,t}{F\,r\,e\,s\,h\,w\,e\,i\,g\,h\,t}\,x\,100$.

### Seed germination rate

Quinoa seeds were treated in order to avoid mold proliferation during the assay with immersion in ethanol 70% for 2 min followed by a wash in bleach 50% with a drop of Tween-20 for 2 min, and then rinsing several times in distilled water (H_2_O). At least, three biological replicates for each cultivar, humidity condition, temperature, and storage time were used with 15 seeds per replicate. The seeds were sown on a wet double layer filter paper [73 g/m2, Filtros Anoia S.A. (Barcelona, Spain)] on Petri dishes. The seeds were transferred to a growth chamber under darkness and at a controlled temperature of 25°C. The percentage of germinated seeds was counted daily for the first week after sowing. Seeds were considered germinated when the radicle protrusion was longer than 2 mm.

### Seed viability

Seed viability tests were performed using the tetrazolium method (2,3,5-triphenyl-2H-tetrazolium chloride, TFT) (44). First, a hundred seeds per replicate were imbibed in distilled water at 30°C for 1 h in order to facilitate longitudinal and superficial cuts of the embryo and to ensure a homogeneous dying of the seed tissues. After cutting, seeds were submerged in 1% TFT at 30°C for 2 h. Seeds with more than 50% of the embryonic tissue stained were considered viable.

### Protein content

The protein content was determined according to AOAC Official Methods (43), measuring nitrogen content with an elemental analyzer (Leco TruSpec) and considering a conversion factor of 6.25 (45).

### Amino acid contents

Amino acid contents were analyzed following the protocol described by Alaiz et al. (46). Briefly, 40 mg of fine-powder ground seeds were hydrolyzed with 4 mL of 6 N HCl using DL-2-aminobutyric acid as the internal standard. The solutions were sealed in tubes under nitrogen atmosphere and incubated in an oven at 110°C for 24 h. A mix of amino acids was used as standard. After digestion, samples were evaporated using a rotary evaporator, and then, the amino acids were resuspended in 25 mL of 1 M borate buffer. Derivatization of amino acids was performed by incubating for 50 min at 50°C, 300 μL of the sample with 6 μL of diethyl ethoxymethylenemalonate diluted to 3 mL in 1 M borate buffer. After samples filtration through a 0.22 μm cellulose filter, amino acids were analyzed by high-performance liquid chromatography coupled to mass spectrometry (HPLC-MS) at Interdepartmental Investigation Service at UAM (SIdI, UAM, Spain). There, the amino acid determination was carried out using HPLC-MS with an Agilent system detector composed of a 1,100 series HPLC coupled to a 6,420 Triple Quadrupole.

### Fat content and fatty acid profile

Fat content was analyzed according to AOAC Official Methods (43). Fatty acid methyl esters from the oil samples were obtained by alkaline treatment using 2N KOH in methanol at room temperature (29). Fatty acid methyl esters separation and quantification gas chromatography were performed according to the European Commission Regulation (EEC) No 2568/91 (47) using an Agilent 6890A Gas Chromatograph (Agilent Technologies, Santa Clara, CA, USA) equipped with a Flame ionization detector (FID) and column Supelco DB-23 60 m × 0.25 mm × 0.25 μm (Agilent Technologies). The results are expressed as content of each fatty acid relative to total seed oil.

### Ferric reducing antioxidant power assay, total flavonoid content, and total phenol content

Seed extracts for the ferric reducing antioxidant power (FRAP) and the total flavonoid content (TFC) analyses were obtained after grinding the seeds to a fine powder and homogenizing 100 mg of the flour in 1 mL of extraction buffer consisting of methanol (50%), acetic acid (1%), and distilled water (49%). These samples were vortexed for 2 min and kept in the dark at 4°C for 48 h, before centrifugation for 15 min at 13500 rpm. The supernatants were stored at –20°C until their use in the FRAP and flavonoid content assays.

The antioxidant capacity of seeds using the FRAP assay was determined following an adaptation of the procedure described by Benzie and Strain (44, 48). FRAP value was expressed as μmol of Fe^2+^/g of seed.

The TFC was determined following the procedure described by Granado-Rodríguez et al. (49). The results were expressed in mg of quercetin equivalents (QE) per gram of quinoa seed (mg QE/g).

To extract total phenols (TPC), 100 mg of seed flour was used in 1 mL of ice-cold methanol (95%). Then, samples were vortexed and centrifuged at 13,500 rpm for 5 min after 48 h kept in the dark and at 4°C. The content of polyphenols was measured following the protocol described by Granado-Rodríguez et al. (44). TPC was expressed as mg of gallic acid equivalents (GAE) per gram of quinoa seed (mg GAE/g).

### Histochemical analysis of quinoa sprouts

Quinoa seeds of both genotypes stored for 12 months without silica gel and at 4 or 37°C were incubated for germination in the dark at 25°C for 3 days. Fifty seedlings per replicate were then incubated under light and at room temperature with either TFT, nitroblue tetrazolium (NBT), or diaminobenzidine (DAB). Staining with TFT 1% for 30 min was used to visualize mitochondrial respiration. For superoxide anion (O^–^_2_) detection, seedlings were stained with 0.5% NBT (w/v) in 2 mM potassium phosphate at pH 7.8 for 30 min. Seedlings were incubated for 5 h with 0.1% DAB in 10 mM Tris-HCl pH 7.5 in order to detect hydrogen peroxide (H_2_O_2_) accumulation. After staining, seedlings were washed, and then observed and photographed using an Olympus SZ61 stereomicroscope (Olympus Corporation, Shinjuku, Tokyo, Japan).

### Statistical analysis

To analyze the influence of the storage temperature, the humidity condition, and the storage time on the seed nutritional quality-related parameters, a three-way ANOVA was performed on each cultivar. Normality and equality of variances of the data were tested through a Kolmogorov–Smirnov’s test and a Levene’s, respectively. For those variables where normality and equal variances could be assumed, a one-way ANOVA test was performed, followed by a Tukey *post hoc* test, to perform multiple comparisons at a probability level of 5% (*p* < 0.05). A Kruskal–Wallis test by ranks was performed when data did not present a normal distribution and a Welch’s ANOVA test followed by a Games-Howell *post hoc* test was performed when variances were not equal, both, at a probability level of 5% (*p* < 0.05). Student’s *T*-test or *U*-Mann–Whitney’s test were carried out when comparing by pairs, when the distribution of data was normal or not, respectively. The SPSS Statistics 26.0 (IBM SPSS Inc., New York, NY, USA) package was used for the statistical analyses. Correlations were analyzed in both genotypes using the Pearson product-moment correlation coefficient (*r*) among all seed parameters. Correlograms were created with the corrplot package (v0.92) (50) running under R (v4.0.2) (51) in RStudio (1.4.1717) (52). At least, three independent biological replicates were used in all the experiments performed.

## Results

### Seed moisture content

**FIGURE 1:**
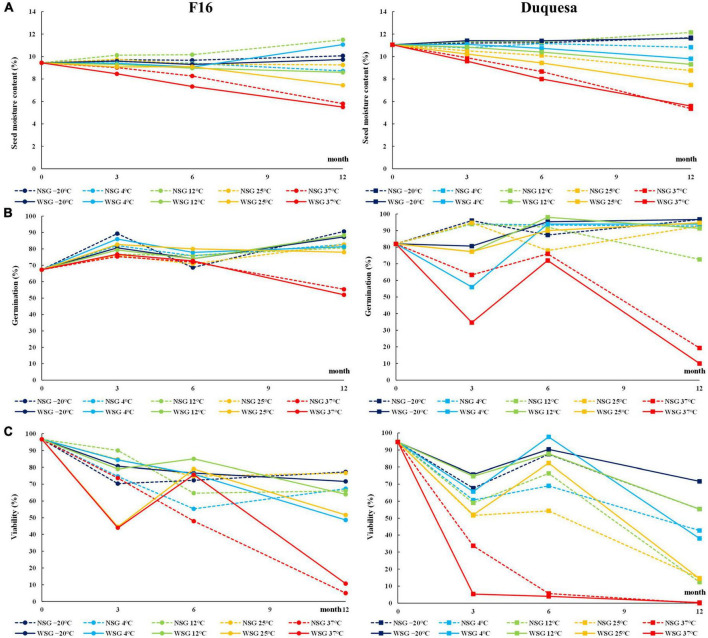
Physiological characteristics of quinoa seeds. **(A)** Evolution of SMC in F16 (left) and Duquesa (right) seeds over time. **(B)** Evolution of germination capacity 1 day after sowing over storage time. **(C)** Viability measured through the TFT method over storage time. NSG, seeds stored without silica gel; WSG, seeds stored with silica gel.

At the beginning of the experiment, the SMC was 9.4 and 11.1% for F16 and Duquesa seeds, respectively. After 12 months of storage, the SMC of F16 ranged between 11.5% (in seeds kept at 12°C without silica gel) and 5.5% (when seeds were kept at 37°C with silica gel), while in Duquesa the SMC ranged between 12.2% (in seeds kept at 12°C without silica gel) and 5.4% (in seeds stored at 37°C with silica gel) (Figure 1A). The changes observed in SMC over time were temperature- and humidity-dependent for both genotypes. Thus, SMC was reduced notably in seeds kept at 37°C (*p* < 0.001 and *p* < 0.001 in F16 and Duquesa, respectively). The same occurred to the Duquesa seeds kept at 25°C after 6 months of storage (*p* = 0.001). The influence of the presence of silica gel was significant for F16 seeds at 3 and 6 months of storage and for Duquesa at 6 and 12 months of storage (*p* < 0.001, *p* = 0.001, *p* = 0.009, and *p* < 0.001, respectively), with a higher SMC found always in seeds kept without silica gel. Moreover, in F16 seeds there was an increase in the moisture content over time when kept at 12°C without silica gel (*p* = 0.023). The moisture remained fairly stable in the rest of cases. In Duquesa, there was a more pronounced decrease over time at all storage temperatures except at –20 and 12°C without silica gel.

### Germination rates and seed viability

The germination capacity after 7 days of incubation of the seeds before storage (time 0) was 91% for F16 and 98% for Duquesa (Supplementary Figure 1). The results showed that the storage caused an increase in the germination capacity over time (*p* < 0.001 and *p* < 0.001 in F16 and Duquesa, respectively) that reached 99% in Duquesa and 96% in F16 seeds, except when seeds were kept at 37°C, which maintained the germination capacity between 86 and 91% after 12 months in F16 (*p* = 0.086) (Supplementary Figure 1). Besides, there was a higher variation in the germination capacity of the seeds after only 1 day of incubation, since storage time and conditions influenced the seed germination, either delaying it or inducing it. In line with this, it was observed that the germination capacity varied depending on the storage time and temperature. Before storage, F16 and Duquesa seeds presented a germination capacity of 67 and 82%, respectively (Figure 1B), while seeds stored for 12 months (excluding those stored at 37°C) presented higher germination rates ranging between 78 and 91% in F16 and between 91 and 95% in Duquesa. However, seeds kept at 37°C showed a decrease in early germination throughout time, dropping to 52% in F16 seeds and to 10% in Duquesa after 12 months storage (*p* < 0.001 and *p* = 0.001, respectively). The humidity condition only factored in the germination rates of Duquesa seeds at 3-month of storage analysis (*p* < 0.001), showing lower early germination rates in seeds kept with silica gel.

Seed viability showed a decrease over time. Viability of seeds before storage were 97 and 95% in F16 and Duquesa seeds, respectively, and dropped over time within a range that varied between 77 to 5% in F16 seeds and between 72 and 0% in Duquesa seeds (Figure 1C). The decrease in seed viability was temperature dependent. In F16 viability were significantly lower in seeds kept at 37°C, decreasing to 44% after 3 months and to 5% after 12 months. Meanwhile, Duquesa seeds stored at 37°C had the lowest viability at all time-points, with rates lower than 4% from the storage month 6 onward. Also, Duquesa seeds kept at 25°C presented lower rates (14% after 12 months, for both humidity conditions) than those kept at –20°C (72%, *p* = 0.001, and 55%, *p* < 0.001, with or without silica gel, respectively, after 12 months). The humidity conditions also affected the viability. In F16, the presence of silica gel had a significant effect on the seed viability (*p* = 0.001) at 6 months storage, maintaining their viability at this time point (or increasing it, as occurred with the F16 seeds stored at 25 and 37°C to later decrease). After 12 months, no differences appeared between seeds stored at the two humidity conditions in F16 (*p* = 0.054). On the other hand, in Duquesa, humidity affected seed viability at 6 and 12 months of storage (*p* = 0.002 and *p* = 0.003, respectively), showing higher rates in the presence of silica gel (*p* = 0.002 and *p* = 0.032, respectively).

### Protein content

**FIGURE 2:**
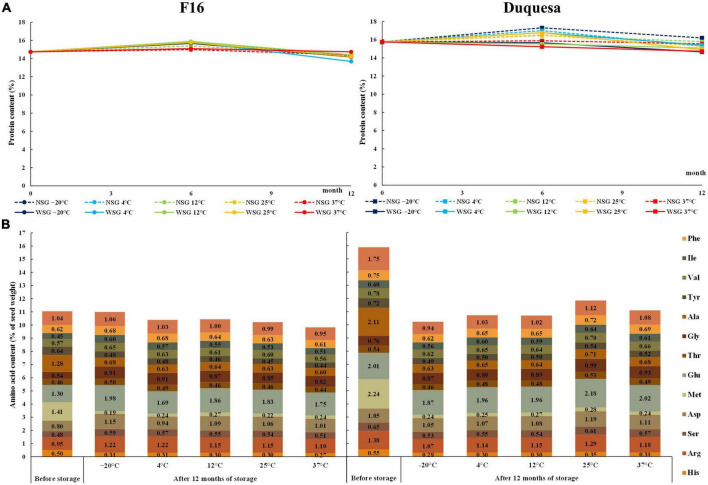
Change in seed protein content and composition over storage time. **(A)** Protein content measured with a nitrogen-to-protein conversion factor of 6.25 in F16 (left) and Duquesa seeds (right) over storage time. NSG: seeds stored without silica gel. WSG: seeds stored with silica gel. **(B)** Amino acid contents expressed as percentage of seed weight in F16 seeds (left) and Duquesa seeds (right) stored without silica gel, measured before storage and after 12 months of storage.

The protein content of seeds before storage was lower in F16 seeds, with 14.74%, than in Duquesa seeds, with 15.75% of seed weight (*p* < 0.001). F16 seeds stored at –20, 4, 12, and 25°C increased their protein content after 6 months to percentages that ranged between 15.38 and 15.88% and dropped after 12 months to contents between 13.68 and 14.41% (*p* < 0.001). Meanwhile, the protein content of seeds stored at 37°C did not show any significant change over time (*p* = 0.143) nor differences with the rest of seeds after 12 months of storage (*p* = 0.402) (Figure 2A). In the case of Duquesa seeds, the humidity conditions of storage impacted the protein contents (*p* = 0.002) (Figure 2A). After 6 months of storage, the protein content in seeds kept without silica gel increased from 15.88 to 17.51% of seed weight, while seeds kept with silica gel, maintained protein contents to values found before storage. After 12 months of storage, the protein contents of both types of seeds decreased varying between 14.66 and 16.19%. Storage temperature also affected Duquesa protein contents after 6 months of storage, showing higher contents in seeds kept at 4 and 25°C than in seeds stored at 37°C (*p* = 0.001).

### Amino acids contents

Before storage, the main amino acids in the amino acid profile of F16 seeds were methionine and glutamic acid (1.41 and 1.30% of seed weight, respectively) (Figure 2B). These were followed by alanine (1.28%), leucine (1.04%), arginine (0.95%) and the aspartic acid (0.8%), and then by the tyrosine (0.64%), phenylalanine (0.62%), valine (0.57%), glycine (0.54%), histidine (0.5%), serine (0.48%), and isoleucine (0.45%). The minor amino acids were cysteine and proline (0.39 and 0.35%, respectively).

Amino acid contents relative to seed weight were overall higher in Duquesa seeds than in F16 seeds since the protein content was also higher (Figure 2B). The amino acid profile was similar, but in proportion, methionine, glutamic acid, and alanine, were more abundant in Duquesa seed protein (*p* = 0.007, *p* = 0.006, and *p* = 0.041, respectively), while histidine, threonine, tyrosine, isoleucine, phenylalanine, proline, cysteine, and the aspartic acid were more abundant in F16 seed protein (*p* = 0.046, *p* = 0.032, *p* = 0.026, *p* = 0.046, *p* = 0.006, *p* = 0.001, *p* = 0.001, and *p* = 0.003, respectively).

After 12 months of storage without silica gel, the contents of histidine, methionine, alanine, and tyrosine significantly decreased in both genotypes at all storage temperatures (*p* < 0.001 in all cases, Figure 2B). There was also a decrease in the content of leucine (except in seeds stored at 37°C), arginine (at –20, 4, and 37°C), serine (at 4, 12, and 37°C), valine (at 4, 12, 25, and 37°C), and in phenylalanine (at 12°C), and an increase glycine (at 4, 25, and 37°C) in Duquesa seeds, although they all increased in proportion. In F16 seeds the glycine and glutamic acid contents increased under all storage temperatures, the isoleucine content increased except in seeds stored at 37°C and the serine content increased in seeds kept at –20 and 4°C. The aspartic acid and arginine increased in all seeds except those stored at 4°C and at 37°C, respectively.

Overall, after 12 months of storage, no differences dependent on the storage temperature were found in Duquesa seeds. However, some amino acids did show changes in F16 seeds. F16 seeds stored at lower temperatures (–20°C, 4°C) showed higher contents of isoleucine (*p* = 0.003), lysine (*p* = 0.007), histidine (*p* = 0.042), serine (*p* = 0.016), and threonine (*p* = 0.009) compared to seeds stored at 37°C. Seeds stored at –20°C showed higher contents of tyrosine (*p* = 0.008) and valine (*p* < 0.001) than seeds stored at 12°C and the opposite trend was observed for methionine (*p* = 0.048).

### Fat content

**FIGURE 3:**
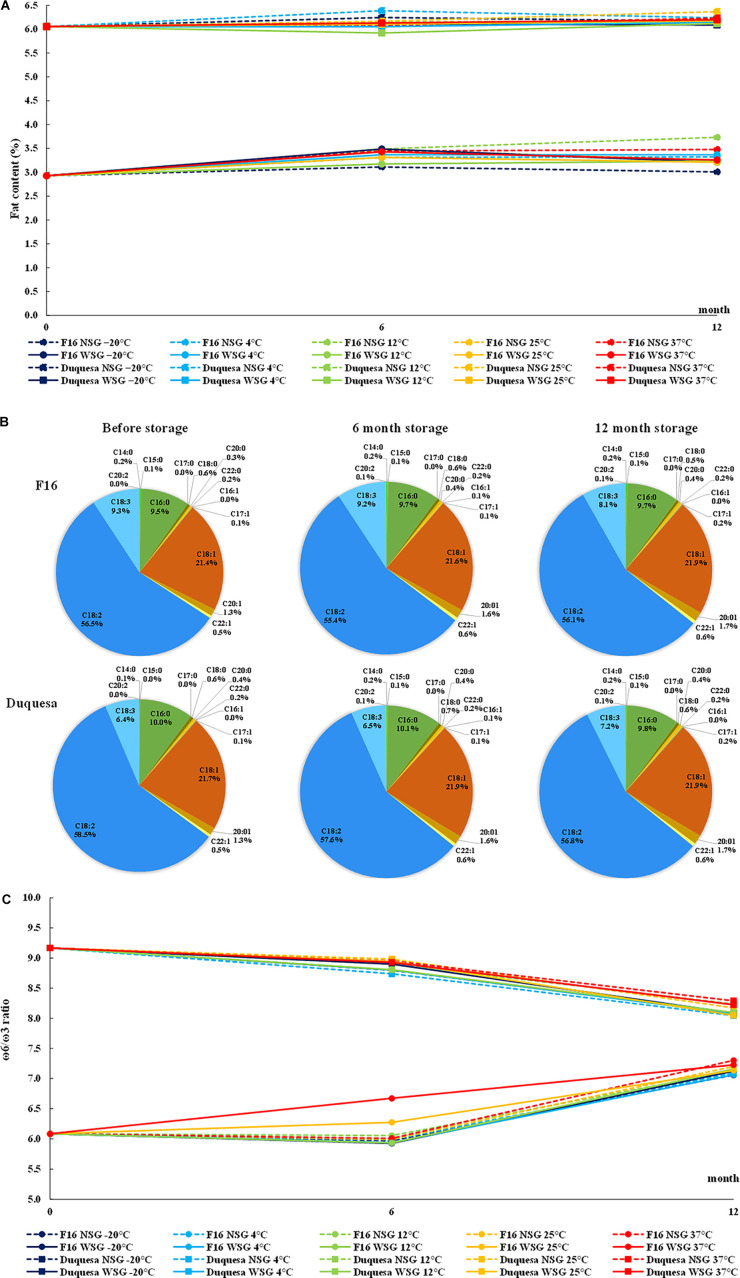
Quinoa fat content and composition. **(A)** Evolution of seed fat content F16 seeds (circles) and Duquesa seeds (squares) stored at different temperatures and humidity conditions. **(B)** Average fatty acid profile F16 seeds (upper row) and Duquesa seeds (lower row) before storage (left), after 6 months of storage (middle), and after 12 months of storage (right). **(C)** ω-6/ω-3 ratio in F16 and Duquesa seeds stored at different temperature and humidity conditions, where ω-6 is the sum of the linoleic and eicosadienoic acid relative contents and ω-3 is the α-linolenic acid relative content. NSG, seeds stored without silica gel; WSG, seeds stored with silica gel; C14:0, myristic acid; C15:0, pentadienoic acid; C16:0, palmitic acid; C17:0, margaric acid; C18:0, stearic acid; C20:0, arachidic acid; C22:0, behenic acid; C16:1, palmitoleic acid; C17:1, margaroleic acid; C18:1, oleic acid; C20:1, gadoleic acid; C22:1, erucic acid; C18:2, linoleic acid; C18:3, α-linolenic acid; C20:2, eicosadienoic acid.

Duquesa seeds showed a total of 6.05% of fat content, more than two-fold the content found in F16 seeds (with a 2.93% fat content of seed weight) (Figure 3A). In F16 seeds, the fat content increased after 6 months and stayed within a range of 3.01–3.73% after 12 months (*p* < 0.001). On the other hand, Duquesa seeds did not experience any statistically significant change over the storage period (*p* = 0.547). The storage temperature and the humidity conditions were not determinant factors in the fat content variation of neither F16 seeds (*p* = 0.988, after 6 months of storage, and *p* = 0.22, after 12 months of storage) nor Duquesa seeds (*p* = 0.0994 and *p* > 0.999, respectively).

Moreover, the fatty acid profile was analyzed for both types of seeds (Figure 3B). In the first analysis, the main fatty acid in quinoa seeds was the linoleic acid, although it showed a higher content in Duquesa seeds (58.49%) than in F16 seeds (56.55%) (*p* < 0.001). In relative abundance, the linoleic acid was followed by the oleic acid, with relative contents of 21.43 and 21.72% in F16 and Duquesa seeds, respectively, and then the palmitic acid (9.48 and 9.98%, respectively), and the α-linolenic acid, which appeared in a higher proportion in F16 seeds (9.29%) than in Duquesa seeds (6.38%). In a lower relative content appeared the gadoleic acid (1.35% in both types of seeds), the stearic acid (0.56 and 0.52% in F16 and Duquesa seeds, respectively), the erucic acid (0.51 and 0.52%, respectively), the arachidic acid (0.27 and 0.36%, respectively). Other minor fatty acids found were the behenic acid (0.18 and 0.17% in F16 and Duquesa seeds, respectively), the myristic acid (0.2 and 0.12%, respectively), the margaroleic acid (0.3 and 0.15%, respectively), the pentadecanoic acid (0.06 and 0.05%, respectively).

With the exception of the linoleic acid in F16 seeds (*p* = 0.150), and the myristic and α-linolenic acids in Duquesa seeds (*p* = 0.159 and *p* = 0.087, respectively), every fatty acid underwent significant changes in its relative content during the storage period. The relative contents of margaroleic acid (*p* < 0.001), oleic acid (*p* < 0.001), arachidic acid (*p* < 0.001), gadoleic acid (*p* < 0.001), behenic acid (*p* = 0.030 and *p* < 0.001 in F16 and Duquesa, respectively), and the erucic acid (*p* = 0.011 and *p* < 0.001, respectively) increased along the time of storage in both types of seeds. The palmitic acid relative content increased in the case of F16 seeds (*p* = 0.004), while it decreased in Duquesa seeds (*p* = 0.004). The content of stearic acid increased in both types of seeds after 6 months of storage, but it was reduced again after 12 months of storage (*p* = 0.003 and *p* < 0.001 in F16 and Duquesa, respectively), and the same happened in F16 seeds in the case of the myristic acid and the pentadecanoic acid (*p* < 0.001 in both cases). Furthermore, the contents of linoleic acid in Duquesa seeds and α-linolenic acid in F16 seeds were reduced after 12 months of storage (*p* < 0.001). The eicosadienoic, palmitoleic, and margaric acids appeared in F16 seeds after 6 months of storage (0.14, 0.05, and 0.02% of the fatty acid total contents, respectively), and they also increased in Duquesa seeds (0.13, 0.06, and 0.04%, respectively). After 12 months of storage these levels remained in the case of the eicosadienoic acid (0.13% in both types of seeds), decreased in the palmitoleic acid (0.046 and 0.044% in F16 and Duquesa seeds, respectively), and kept increasing in the case of the margaric acid (0.041% in both types of seeds).

Overall, the total of mono-unsaturated fatty acids (MUFA, palmitoleic, margaroleic, oleic, gadoleic, and erucic acids) increased along the time of storage, while the polyunsaturated fatty acids (PUFA, linoleic, α-linolenic, and eicosadienoic acids) were reduced. The saturated fatty acids (SFA, myristic, pentadienoic, palmitic, stearic, arachidic, and behenic acids) increased after 6 months of storage in both types of seeds and stayed up in the case of F16 seeds but decreased again after 12 months of storage in the case of Duquesa seeds.

The humidity and temperature conditions of storage did not affect the lipidic profile until after 12 months of storage. The use of silica gel impacted the relative content of the palmitoleic (*p* < 0.001 and *p* = 0.001 in F16 and Duquesa, respectively), margaric (*p* < 0.001), and erucic acids (*p* = 0.022 and *p* = 0.003, respectively) in both types of seeds and the eicosadienoic acid in Duquesa seeds (*p* = 0.001), being the relative contents higher in seeds kept without silica gel. In the case of the gadoleic acid in F16 seeds, however, the contents were higher in seeds stored with silica gel (*p* = 0.040). Cool storage temperatures (–20, 4, and 12°C) induced higher contents of margaric acid in both F16 and Duquesa seeds (*p* = 0.004 and *p* = 0.003, respectively) and of erucic acid in Duquesa seeds (*p* = 0.003) than warm storage temperatures (25 and 37°C), and lower contents of oleic acid in F16 seeds (*p* = 0.008).

The ω-6/ω-3 ratio (linoleic and eicosadienoic acids/α-linolenic acid) was also calculated. The ratio was lower in F16 seeds, with an initial value of 6.1:1, than in Duquesa seeds, with a ratio of 9.2:1 (Figure 3C). In F16 the ratio increased to a level of between 7.0:1 and 7.3:1 after 12 months of storage, while in Duquesa seeds the ratio decreased to a range from 8.0:1 to 8.3:1. The storage conditions did not significantly impact the ratio in either case (*p* = 1).

### Antioxidant capacity

**FIGURE 4:**
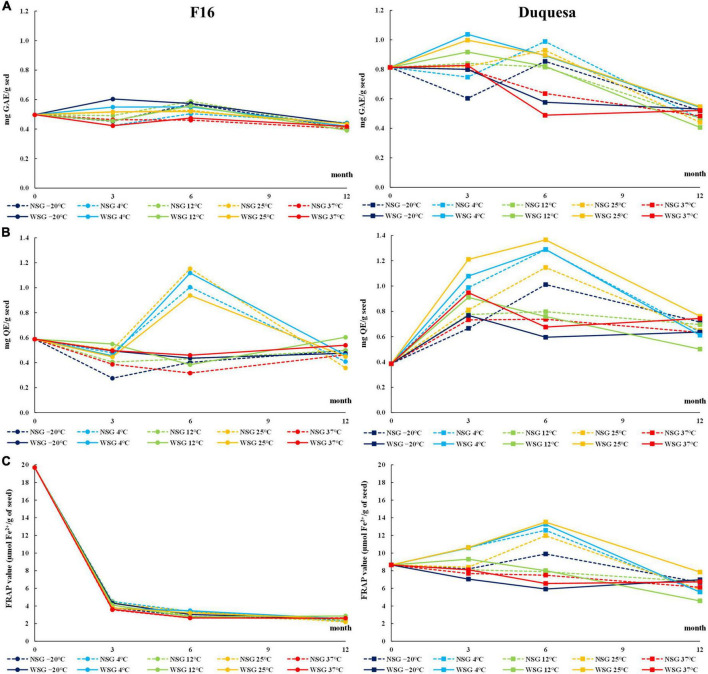
Antioxidant evolution over storage time. **(A)** Total phenolic contents (TPC) over storage time in F16 (left) and Duquesa seeds (right), measured in gallic acid equivalents (GAE) through the Folin-Ciocalteu method. **(B)** Total flavonoid content (TFC) measured in quercetin equivalents (QE) through the AlCl_3_ method. **(C)** Antioxidant capacity of quinoa seeds measured through the FRAP assay expressed as amount of Fe^2+^ ions reduced in seeds. NSG, seeds stored without silica gel; WSG, seeds stored with silica gel.

The phenol content (TPC) of seeds before storage was higher in Duquesa (0.81 mg GAE/g seed) than in F16 seeds (0.50 mg GAE/g seed) (Figure 4A).

In F16 seeds, the TPC changed over time (*p* < 0.001), increasing after 6 months of storage to levels ranging between 0.48 and 0.57 mg GAE/g seed, and then dropping to levels between 0.39 and 0.44 mg GAE/g seed after 12 months of storage. Seeds stored with silica gel were the ones that showed higher TPC (*p* = 0.034). At 6 months of storage, temperature was the factor that determined changes in TPC (*p* < 0.001), being the TPC highest in seeds kept at –20 and 12°C, and lowest in seeds kept at 37°C. The variation in TPC was overall small in F16 seeds compared to Duquesa (Figure 4A).

In Duquesa seeds there was an important influence of the humidity conditions (*p* < 0.001 and *p* = 0.002 after 3 and 6 months, respectively) and temperature (*p* < 0.001 after 3 and 6 months) during their storage. Seeds kept with silica gel increased their TPC after 3 months ranging between 0.83 and 1.04 mg GAE/g seed, and then dropped after 6 months to levels between 0.49 and 0.90 mg GAE/g seed, while seeds stored without silica gel remained within a range between 0.60 and 0.99mg GAE/g seed. At these time-points (3 and 6 months of storage), TPC was higher in seeds stored at 25 and 4°C than in seeds kept at –20°C, and after 6 months of storage, the lowest TPC belonged to seeds kept at 37°C. After 12 months of storage, the TPC of Duquesa seeds, at both humidity conditions, dropped to levels ranging between 0.41 and 0.55 mg GAE/g seed (Figure 4A).

Regarding the TFC, in both, F16 and Duquesa, there was a clear influence of the storage temperature after 3 months (*p* = 0.043 and *p* < 0.001, respectively) and 6 months (*p* < 0.001 in both F16 and Duquesa seeds) of storage. These differences in TFC allowed us to classify the seeds into two groups, those kept at 4 and 25°C, and those stored at –20, 12, and 37°C.

In F16 seeds, the TFC of seeds before storage was 0.59 mg QE/g seed. Seeds kept at –20, 12, and 37°C significantly decreased their TFC (*p* < 0.001) after 3 months of storage, while seeds kept at 4 and 25°C had a significant increase in TFC after 6 months of storage to levels that varied from 0.94 to 1.15 mg QE/g seed. After 12 months of storage, there was a decrease in the TFC of seeds kept at 4 and 25°C, while the TFC of seeds stored at –20, 12, and 37°C increased, resulting in similar levels in both groups ranging from 0.32 to 0.46 mg QE/g seed, lower than the initial TFC (Figure 4B).

In Duquesa seeds, the TFC before storage was 0.39 mg QE/g seed, lower than in F16 seeds (Figure 4B). After 3 months of storage, the TFC increased to levels that ranged between 0.6 and 0.95 mg QE/g seed in seeds stored at –20, 12, and 37°C, and to levels between 0.81 and 1.21 mg QE/g seed in seeds kept at 4 and 25°C. The latter group kept increasing the TFC to levels between 1.15 and 1.36 mg QE/g seed after 6 months of storage, while the former group maintained the TFC between 0.6 and 1.01 mg QE/g seed. After 12 months, seeds kept at all temperatures decreased their TFC to levels between 0.5 and 0.76 mg QE/g seed, although the content was higher than the levels found before storage.

In both, F16 and Duquesa, packaging with or without silica gel had an influence on the TFC levels after 3 months of storage (*p* = 0.001 and *p* < 0.001, respectively), with higher TFC in seeds kept with silica gel.

The FRAP value (μmol Fe^2+^/g seed) was determined to evaluate the seed antioxidant capacity. The values before storage were 19.7 in F16 and plummeted to levels between 3.56 and 4.51 after 3 months of storage (Figure 4C). Then, the FRAP value kept decreasing (*p* < 0.001) until reaching levels ranging from 2.52 to 2.87 after 12 months of storage. The storage temperature significantly affected the antioxidant capacity (*p* < 0.001) and produced lower FRAP values in seeds kept at 37°C. In Duquesa seeds, the FRAP value before storage was 8.65, lower than the initial antioxidant capacity of F16 seeds. After 3 and 6 months of storage, there was a significant influence of the temperature in the FRAP values (*p* < 0.001), being higher in seeds stored at 4 and 25°C compared to the seeds stored at –20, 12, or 37°C. The FRAP values of seeds stored at 4 and 25°C increased until reaching values from 11.97 to 13.51 after 6 months of storage and then decreased to a range from 5.58 to 7.84 after 12 months of storage. In the case of seeds stored at –20, 12, and 37°C, the FRAP values had a steady decrease over time until reaching similar ranges compared to the rest of Duquesa seeds after 12 months of storage (Figure 4C). In Duquesa seeds, there was also a difference between seeds kept with silica gel, which had higher FRAP values after 3 months of storage, and seeds kept without silica gel (*p* = 0.046).

### Oxidative status of quinoa sprouts

**FIGURE 5:**
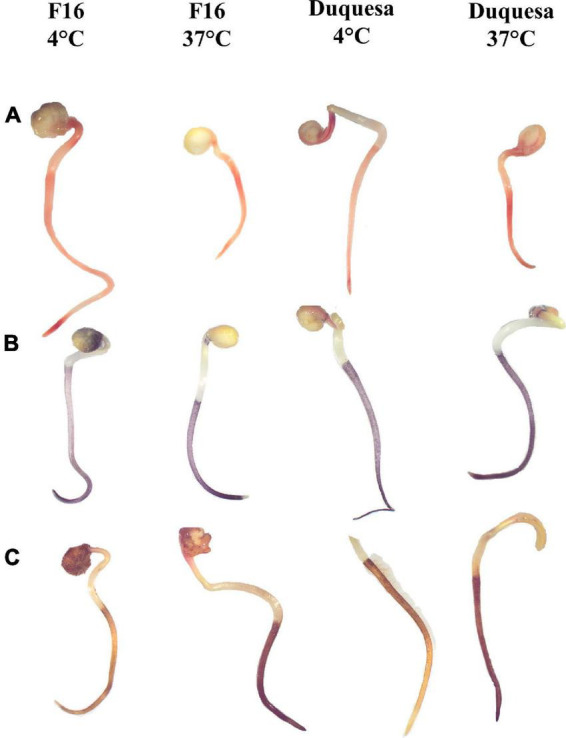
Staining of quinoa sprout from seeds stored for 12 months. **(A)** Staining with TFT, indicating metabolic activity, **(B)** NBT, indicating superoxide anion accumulation, and **(C)** DAB, showing hydrogen peroxide accumulation, of F16 seedlings stored at 4 and 37°C and Duquesa seeds stored at 4 and 37°C, from left to right, respectively. The black bar indicates scale of 5 mm.

Sprouts of quinoa seeds stored for 12 months at 4 and 37°C were stained with TFT, NBT, and DAB to evaluate the impact of the storage temperature on the metabolic activity (TFT) and reactive oxygen species generation (NBT and DAB). F16 seeds stored at 4 and 37°C stained with TFT showed an orange coloration in both the root and the hypocotyl (Figure 5A and Supplementary Figure 2). In seeds stored at 4°C, the staining was more intense in roots than in hypocotyls, and the root presented a dark red color in the meristem and in the transition to the hypocotyl. In F16 seeds stored at 37°C, the root color was not as intense or different from the hypocotyl staining. In Duquesa seedlings the reddish staining was more intense in roots of seedlings stored at both temperatures and the hypocotyl was not as colored as in F16 seedlings (Figure 5A).

Nitroblue tetrazolium staining showed a dark blue coloring localized in the roots. Differences were found in the intensity of the staining when comparing between storage temperatures, being the sprouts of seeds stored at 37°C the ones with a more intense coloring (Figure 5B and Supplementary Figure 3).

When staining seedlings with DAB, the stain was localized along the roots, including the root hairs. The color ranged from light yellow when roots were not well stained, to amber, to dark brown when the staining was more intense, indicating higher accumulation of H_2_O_2_. In F16 sprouts whose seeds were kept at 4°C, the most common pattern of coloring was amber roots with the upper section often darker, while in sprouts of seeds kept at 37°C the coloring was homogenous and dark brown (Figure 5C and Supplementary Figure 4). In Duquesa seedlings, roots staining was equally intense along the root in the case of sprouts of seeds stored at 37°C, and darker than sprouts of seeds stored at 4°C, although these roots were darker than those from F16 seeds stored at 4°C (Figure 5C and Supplementary Figure 4).

### Correlations and principal components analysis

**FIGURE 6:**
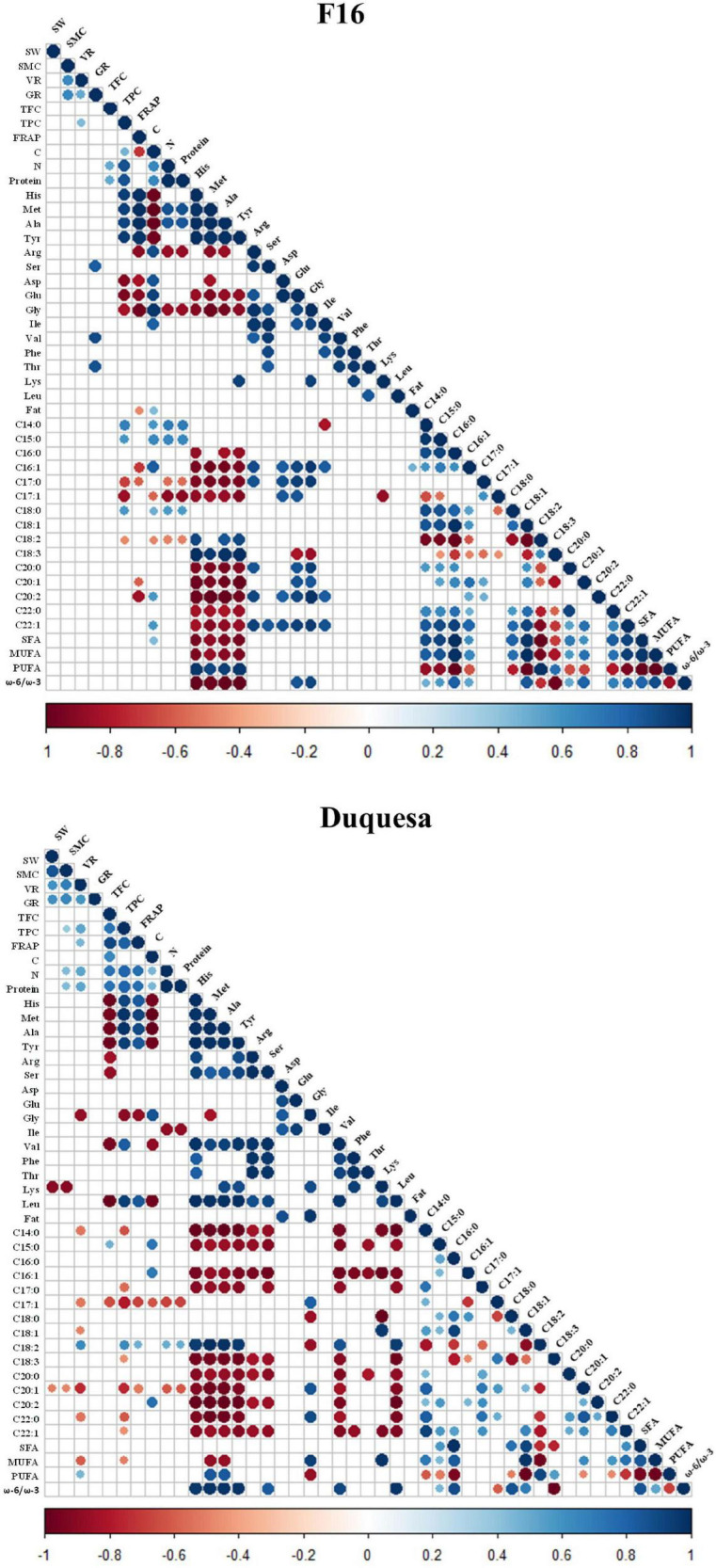
Correlogram of quinoa seed quality variables. *r* coefficient of correlations of variables measured in F16 **(left)** and Duquesa **(right)** seeds, with a significance of *p* < 0.05. Blue circles show positive correlations while red circles indicate negative correlations. SW, 1000 seeds weight; SMC, seed moisture content; VR, viability rate; GR, germination 1 date after sowing; TPC, total phenolic content; FRAP, antioxidant power; C, carbon content; N, nitrogen content; C14:0, myristic acid relative content; C15:0, pentadienoic acid relative content; C16:0, palmitic acid relative content; C16:1, palmitoleic acid relative content; C17:0, margaric acid relative content; C17:1, margaroleic acid relative content; C18:0, stearic acid relative content; C18:1, oleic acid relative content; C18:2, linoleic acid relative content; C18:3, α-linolenic acid relative content; C20:0, arachidic acid relative content; C20:1, gadoleic acid relative content; C20:2, eicosadienoic acid relative content; C22:0, behenic acid relative content; C22:1, erucic acid relative content.

A Pearson’s correlation coefficient test was performed to analyze the correlation between seed quality variables (Figure 6 and Supplementary Figure 5).

In F16 seeds, there was a strong positive correlation between the SMC and the seed viability and fat content (*r* = *0.64* with both parameters). The viability was also positively correlated with the germination capacity after 1 day and the phenolic content (*r* = 0.64 and *r* = 0.43, respectively). The protein content correlated positively with the phenolic and flavonoid contents (*r* = 0.87 and *r* = 0.51, respectively) and with the carbon content (*r* = 0.64). There was a first group of amino acids which included histidine, methionine, alanine, and tyrosine contents, that presented a strong positive correlation among them (*r* > 0.97) and with the antioxidant capacity and contents of phenols, protein, and the linoleic and α-linolenic acids and the sum of PUFA contents (Figure 6). This group also correlated negatively with the content of carbon, and the group of fatty acids containing the palmitic, palmitoleic, margaric, margaroleic, arachidic, gadoleic, eicosadienoic, behenic, and erucic acids relative contents, as well as with the sum of the SFA and MUFA contents and the ω-6/ω-3 ratio. It also correlated negatively with the contents of a second group of amino acids that included arginine, aspartic and glutamic acid, and glycine. This second group of amino acids correlated positively among them and with the carbon content, the contents of the amino acids isoleucine, valine, and the contents of the aforementioned group of fatty acids, and negatively, with the protein and α-linolenic acid contents (Figure 6). In the case of the fatty acids, there was a strong positive correlation between the myristic, pentadienoic, palmitic, palmitoleic, stearic, oleic arachidic, gadoleic, eicosadienoic, behenic, and erucic acid contents, and, these, positively correlated with the phenolic, carbon, protein and the second group of amino acids contents and the ω-6/ω-3 ratio, and negatively with the first group of amino acids contents and the linoleic and the α-linolenic acid contents. The linoleic and the α-linolenic acid contents correlated positively with each other (*r* = 0.63) and with the sum of PUFA contents (*r* = 0.98 and *r* = 0.76, respectively) and the ω-6/ω-3 ratio (*r* = 0.74 and *r* = 0.98, respectively) and the contents of the first group of amino acids, and, negatively, with the contents of phenolic compounds, carbon, protein, glutamic acid, glycine, and the rest of fatty acids and the sum of SFA and MUFA. The margaric and margaroleic acid contents correlated positively with each other (*r* = 0.61) and with the second group of amino acids contents and, negatively, with the contents of phenolic compounds, carbon, protein, the first group of amino acids, and the α-linolenic acid.

In Duquesa seeds, the physiological parameters (including seed weight, SMC, and viability) correlated positively among them (*r* > 0.59) and with the germination rate and the phenolic and protein contents, and, negatively, with the lysine and gadoleic acid contents. The antioxidant contents (which include the total phenol and flavonoid contents) and capacity (FRAP) strongly and positively correlated among them (*r* > 0.74) and with the protein and linoleic acid contents, and, negatively, with the glycine, margaroleic, and gadoleic contents. The protein content also correlated positively with the linoleic acid content, and, negatively, with the isoleucine, margaroleic, and gadoleic contents. Most amino acid contents (including histidine, methionine, alanine, tyrosine, arginine, serine, valine, phenylalanine, threonine, and leucine) showed a positive correlation among them (which was strong), and with the linoleic acid content and the ω-6/ω-3 ratio, and negative with the flavonoid, pentadienoic and α-linolenic acid and carbon contents, and the group of fatty acids formed by the myristic, palmitoleic, margaric, arachidic, gadoleic, eicosadienoic, behenic, and erucic acids (Figure 6). This group of fatty acids showed positive correlations amongst them and with the margaroleic, stearic, and oleic acids contents and the sum of MUFAs, and negative correlations with the viability, the phenolic compounds, linoleic and linolenic acids, and the PUFA contents. The contents of linoleic acid and α-linolenic acid strongly and positively correlated with the sum of PUFAs contents (*r* = 0.91 and *r* = 0.55, respectively) but did not show a significant correlation with each other. While the linoleic acid correlated positively with the viability, the antioxidant capacity and the contents of phenolic contents, the protein, histidine, methionine, alanine, tyrosine, valine, and leucine, and negatively with the glycine, myristic, palmitic, margaric, oleic, gadoleic, behenic, and erucic acids contents, the α-linolenic acid correlated negatively with those amino acids, the phenolic content, and the palmitic, palmitoleic, stearic, and oleic acids, the SFA sum and the ω-6/ω-3 ratio.

## Discussion

Storage is the most critical process for seed preservation after harvesting, with estimated losses of up to 60% of seeds in developing countries, where small farmers are still dependent on traditional storage methods (53, 54). These account for both, physical and quality losses of the product. However, these losses are preventable and could be avoided by using more appropriate storage methods that allow the control of the environmental conditions (55). Up to date, it is widely known that factors such as the storage temperature, SMC (in response to RH), and oxygen availability, are the main environmental factors driving seed aging during storage (5). However, fewer studies have focused on analyzing their impact on seed nutritional quality particularly in quinoa. In the present study, both seed viability and nutritional parameters were evaluated in seeds of two genotypes of the emerging crop quinoa stored at different temperature and humidity conditions.

Harrington (6) postulated a general rule for orthodox seeds: for every 5°C decrease in storage temperature, as well as for every 1% decrease in SMC (raging between 5 and 14%), seed longevity can be doubled. Above 14% SMC, metabolic and fungal activities can be induced, causing spoilage, and, in the case of quinoa seeds, longevity could be gained with lower SMC until reaching a minimum of 4.1% (56). In the current study, the initial SMC was 9.4 and 11.1% in F16 and Duquesa seeds, respectively (Figure 1A), falling into the appropriate range to maintain seed viability and avoid spoilage (11, 12). Quinoa seeds are highly hygroscopic due to the porosity of their integuments, which results in quick absorption and desorption of water (57). This characteristic causes problems in seeds stored using traditional methods, especially in countries with humid monsoon season, that results in high RH (8). In the present study, the use of hermetic bags prevented the gain of moisture. In fact, SMC was maintained throughout storage at cold temperatures but decreased at elevated temperatures in both genotypes, being steeper in Duquesa seeds in the presence of silica gel (Figure 1A). The silica gel impacted SMC by capturing moisture, while high temperatures (i.e., 37°C) prompted a rapid loss of moisture (Figure 1A), which can be explained by the activation of cellular respiration, as suggested in previous works performed in quinoa (12). Although SMC is a very important factor in determining seed quality during storage (8), in the present study it was not the primary factor determining seed quality as it was maintained within an adequate range in both genotypes at all times (Figure 1A).

According to Harrington’s rule (1972), the decrease in SMC observed in quinoa seeds would have increased their longevity while the high storage temperatures would have caused a decrease. This was shown in both quinoa genotypes throughout storage, with germination rates ranging between 90 and 97% in F16 and Duquesa seeds, respectively, except for F16 seeds stored at 25 and 37°C, in which germination rates decreased below 90% (Supplementary Figure 1). Other studies have reported decreases in the germination capacity when seeds were stored at temperatures higher than 30°C (58, 59), and also at room temperature in quinoa seeds (40, 60), although the negative effect at this temperature seems to be dependent on genotype (61). Interestingly, the germination rate was also accelerated over time in both quinoa genotypes, with increments in germination rates after 1 day observed in all seeds except those stored at 37°C (Figure 1B). This was observed previously in quinoa seeds stored at low temperatures (39) and could be related to the release of secondary dormancy, a common strategy in spring-summer crops to ensure germination after the low winter temperatures (41, 62, 63).

Viability also showed an important decrease in both genotypes and all storage conditions throughout the storage time (Figure 1C). This phenomenon, called seed aging, is known to be accelerated under inappropriate storage conditions (like high SMC or elevated storage temperatures) (5, 58, 59), as observed in the present study at 25 and 37°C (Figure 1C). Also, the accumulation of reactive oxygen species (ROS) has been considered as one of the main seed intrinsic factors determining seed aging (64–66). During the storage of dry seeds, low moisture contents limit enzymatic metabolism, preventing ROS formation via cellular respiration. However, this can also inhibit the antioxidant machinery, leading to a gradual accumulation of ROS, eventually causing aging (64, 65).

In line with this, histochemical staining methods were used in the present study in 12-month storage sprouts in order to confirm if an accumulation of ROS was related to the loss of seed viability and the decrease and delay of germination in seeds stored at 37°C (Figures 1B,C and Supplementary Figure 1). TFT staining yielded similar results in both quinoa genotypes and both storage temperatures tested, 4 and 37°C, indicating that seedlings had comparable metabolic activities while germinating (Figure 5A). However, sprouts of seeds stored at 37°C showed more intense coloring than the sprouts of seeds stored at 4°C for both NBT and DAB staining (Figures 5B,C), indicating an accumulation of the ROS superoxide anion (O^–^_2_) and hydrogen peroxide (H_2_O_2_), respectively (67). This increased ROS accumulation under high storage temperatures fits well with the fact that heat stress has been proven to increase oxidative stress and inhibit antioxidant activity (68). Quinoa seed loss of viability has been previously related to a higher concentration of products of Maillard’s reactions (61), and lipid peroxidation (8), both processes induced by oxidative stress. Since ROS accumulation showed differences related to the storage conditions, this suggests that their effects on macromolecules, and thus, on their nutritional characteristics, should also be temperature- and/or humidity- dependent.

As previously mentioned, oxidative stress can cause lipid peroxidation, which results in the degradation of unsaturated fatty acids producing free radicals that contribute to oxidative stress (69), which in turn can result in rancid odors and flavors in the seeds, decreasing their shelf life affecting their monetary value (70). Indeed, lipid peroxidation targets primarily PUFAs (64), which are of great importance for human health since diets rich in unsaturated fatty acids can contribute to cardiovascular disease prevention (71). In the present study, there was a decrease in the relative content of PUFA over storage time in both quinoa genotypes (which also meant a relative increase in the MUFA content), although the overall decline was very small (Figure 3B). According to the high levels of fat found in the present and previous studies (29, 72, 73), elevated rates of peroxidation would have been expected. However, quinoa seeds have shown a resistance to ROS-induced peroxidation, probably due to a strong antioxidant capacity (70). Also, although the decrease in PUFAs relative content was significant at the seed oil level, this did not significantly affect the total fat content (Figure 3A), which in fact showed an increase after 6 months in F16 seeds (not significant in Duquesa seeds), probably due to the decrease in total seed weight after the loss of SMC (Figure 1A). Interestingly, PUFAs decline differed between genotypes. The different evolution patterns of fat content between genotypes were expected, since the initial content and the fatty acid profile were also very different (Figures 3A,B), as previously reported when studying different quinoa genotypes (29). In F16, the targeted fatty acid was α-linolenic acid (C18:3), while in Duquesa seeds there was a reduction of linoleic acid (C18:2) over time, the main fatty acid present in quinoa seeds (Figure 3B) (29, 73). The relation between these two fatty acids is also important in human diets, since they are the main ω-6 (linoleic acid) and ω-3 (α-linolenic acid) found in our diet and they can impact the ω-6/ω-3 ratio, which is recommended to be between 5:1 and 10:1 in order to reduce the risk of cancer and cardiovascular diseases (74). In the present study, the ω-6/ω-3 ratio of both genotypes fell within the recommended range for daily intake, being F16 oil closer to the optimum.

Reactive lipid radicals produced during the lipid peroxidation can interact with other molecules, like proteins and nucleic acids, causing further nutritional quality loss in stored quinoa seeds (14, 61). For instance, lipid aldehydes can interact with proteins forming complex high-molecular weight aggregates with low solubility through the Maillard reactions (75). The formation of these aggregates is sensitive to high moisture contents and to storage temperatures above 30°C (75) and entails a loss of function in proteins, leading to a decrease in the metabolic capacity and thus, in seed viability (61) (Figure 1C). These reactions are also associated with protein carbonylation that results in protein degradation (14, 76) and, probably, also in altered protein digestibility, as previously found in maize or rice grains (14, 77, 78). In this study, an increase and decrease in protein contents after 6 and 12 months, respectively, were observed (Figure 2A), as was previously seen in stored quinoa seeds (12). There are probably two different phenomena at play in this regard. First, the decrease in the seed weight caused by the moisture loss over time is bound to impact the relative contents of other molecules, like the relative protein content, which would explain the increase after six months or the lack of decrease in F16 seeds after 12 months of storage at 37°C, which showed lower SMC (Figure 1A). Second, the degradation of protein into small peptides and amino acids through Maillard reactions (61).

Nonetheless, although protein degradation may occur, the total contents of amino acids do not necessarily have to change. It is known that certain amino acids are more susceptible to oxidation in Maillard reactions, like histidine, the aromatic amino acids, including tyrosine, and the sulfur-containing amino acids, like methionine (79). Thus, the decline of histidine, tyrosine, and methionine over storage time found in both quinoa genotypes (Figure 2B) could be related to these reactions, especially when considering the positive correlation found between these amino acids and the PUFA and antioxidant contents in both quinoa genotypes (Figure 6). Furthermore, the decrease found in F16 seeds stored at 37°C compared to the lowest temperatures, 4°C and –20°C (Figure 2B), could be explained by the action of dehydratases and desulfhydrases on the mitochondrial matrix in the case that high temperatures activate mitochondrial respiration (80). Still, little is known about the mechanisms responsible for the amino acid variations observed in seeds or grains during storage and further research is needed to clarify this aspect (81). Overall, the essential amino acid contents stayed close to the recommended by the FAO (82).

Quinoa seeds have an overall good antioxidant system (83) that makes them more stable over storage time (70) and also contributes to their nutritional quality and health benefits, reducing the risk of oxidative stress-related chronic diseases like cancer, cardiovascular disease, or diabetes (31). The antioxidant system in seeds is constituted by antioxidant compounds, such as vitamin E, polyphenols, and flavonoids, while the antioxidant enzymes cannot activate their function until germination due to the low moisture content. These antioxidant compounds work to regulate the concentration of ROS in cells, but may not be enough to prevent ROS accumulation and oxidative stress when seeds are stored in inadequate conditions (65). Thus, during oxidative stress, there is a reduction in antioxidant compounds, since they are used as free-radical scavengers (70). In the present study, a reduction of polyphenols content and antioxidant activity was observed over time in both quinoa genotypes, steeper in seeds stored at 37°C (Figure 4), and a positive correlation with the linoleic acid relative content was present (in Duquesa, Figure 6), supporting the antioxidant role in preventing degradation of fatty acids and with a positive effect in seed viability (Figure 6). This antioxidant components depletion and reduction in the antioxidant capacity when facing oxidative stress was already known to occur in quinoa seeds (84) and is a common response to oxidative stress caused by seed aging (85). Interestingly, remarkable differences were found between genotypes when analyzing these antioxidant compounds and antioxidant activities during storage. Generally, Duquesa seeds showed greater variations with temperature, but kept larger antioxidant values than F16 seeds, which drastically decreased their antioxidant capacity after 3 months of storage (Figure 4C). These genotypic variations can be related to the differences in the antioxidant contents normally observed among quinoa cultivars (49, 86). On the other hand, phenols (including flavonoids) are expected to vary during storage, not only due to their role in detoxifying ROS but also because mature seeds suffer biochemical changes (i.e., lignification) to ensure impermeabilization as protectant mechanisms to ensure seed preservation, in which phenols are directly implicated (87). Nonetheless, further research should be performed to elucidate the exact processes responsible for the antioxidant variations.

Overall, F16 and Duquesa seeds showed significant differences in every quality parameter measured before storage. This is an example of the great variability that has been observed previously among different quinoa genotypes (49). Furthermore, although the general trends in quality changes were similar for both genotypes, some differences could be found in the viability and germination capacity the antioxidant capacity, and the opposing degradation of α-linolenic acid in F16 seeds in contrast to the decrease in linoleic acid contents observed in Duquesa seeds.

The storage temperature proved to be determinant for most nutritional quality traits studied. Thus, special attention must be paid to this aspect when storing quinoa seeds. It is very important for quinoa seed quality to avoid high temperatures during storage, which could be reached if seeds are kept in unventilated rooms in hot climates (88–90). On the other hand, seed storage at room temperature (25°C) did not negatively affect the nutritional traits studied, although it should also be avoided to prevent viability loss. Storage of seeds at cold controlled temperatures (–20, 4, or 12°C) has been reported to be able to maintain the germination capacity and nutritional quality in quinoa seeds (12, 39, 40, 60), as happened in the present study, but the most favorable storage temperature according to the results here presented is 4°C, especially after 6 months of storage. Thus, the recommended storage conditions according to the present findings are a storage temperature of 4°C in hermetic bags, if the initial SMC is 12% or lower. Since quinoa is a crop still largely produced at small farms and household levels in South America (91–93), the habit of drying seeds before storage (53) and the improvement of storage conditions, investing in storage facilities that allow for colder temperatures or the use of appropriate storage packaging (2) could greatly contribute to improving food safety and livelihoods of quinoa producers. Overall, it is of great importance to follow these recommendations for quinoa storage and to continue to study and optimize storage technologies for this and other alternative crops, since the management during the post-harvest period is fundamental to achieving global food security, especially at trying times in which food production or transport are limited (1).

## Conclusion

In conclusion, the results provided in the present work explored the impact of storage conditions on the physiological and nutritional quality of quinoa seeds. Although the use of silica gel as a desiccant was studied as well as the storage temperature, only the temperature factor proved to be determinant for almost all nutritional and physiological parameters measured, and the effects were often genotype-dependent. It was proved that quinoa seed quality can be maintained and even improved provided that optimal storage conditions are used, keeping SMCs and storage temperatures low. In seeds stored at low temperatures (4 and –20°C), there was an increase in essential amino acids like isoleucine, serine, glycine, glutamic acid, and arginine after 12 months of storage. There were opposite trends in the antioxidant contents and capacity, with higher contents and activity, in seeds stored at 4°C. Going further, the viability of the seeds stayed high at low temperatures, and germination was accelerated, which are very positive features for producers. On the other hand, poor storage conditions are detrimental to seed quality. In quinoa seeds stored at 37°C, an accumulation of ROS was observed, indicating oxidative stress, which probably was related to lower antioxidant contents and capacity and a reduction in the contents of essential amino acids like isoleucine, lysine, histidine, and threonine, resulting in a delayed and reduced germination capacity. Therefore, low storage temperatures (4°C) and the use of hermetic bags to maintain SMC lower than 12% are proposed as key conditions that can be controlled to maximize the nutritional potential of quinoa seeds, increasing the economic value as well as the germinative potential for the next growing season.

## Data availability statement

The original contributions presented in this study are included in the article/Supplementary material, further inquiries can be directed to the corresponding author.

## Author contributions

SG-R: methodology, formal analysis, investigation, writing—original draft, visualization, and project administration. IM-G and JM: investigation and writing—review and editing. MJR and PC: investigation. LH: methodology and writing—review and editing. LB: writing—review and editing. MR: conceptualization, methodology, validation, resources, writing—review and editing, supervision, project administration, and funding acquisition. All authors contributed to the article and approved the submitted version.

## Supplementary material

Supplementary Figure 1 Germination rate of quinoa 7 days after sowing throughout 12 months of storage. NSG, seeds stored without silica gel; WSG, seeds stored with silica gel.

Supplementary Figure 2 2,3,5-triphenyl-2H-tetrazolium chloride staining of quinoa seedlings. Seeds of F16 and Duquesa genotypes stored for 12 months at 4°C or 37°C were grown for 3 days and their sprouts were stained with TFT. Scale bars indicate 5 mm.

Supplementary Figure 3 Nitroblue tetrazolium staining of quinoa seedlings. Seeds of F16 and Duquesa genotypes stored for 12 months at 4 or 37°C were grown for 3 days and their sprouts were stained with NBT. Scale bars indicate 5 mm.

Supplementary Figure 4 Diaminobenzidine staining of quinoa seedlings. Seeds of F16 and Duquesa genotypes stored for 12 months at 4 or 37°C were grown for 3 days and their sprouts were stained with DAB. Scale bars indicate 5 mm.

Supplementary Figure 5 Correlogram of variables measured in F16 and Duquesa seeds. Pearson correlation coefficients (*r*) are given when the correlation between variables is statistically significant (*p* < 0.05). Red coefficients indicate negative correlations and blue numbers show positive correlations. SW, 1000 seeds weight; SMC, seed moisture content; VR, viability rate; GR, germination 1 date after sowing; TPC, total phenolic content; FRAP, antioxidant power; C, carbon content; N, nitrogen content; C14:0, myristic acid relative content; C15:0, pentadienoic acid relative content; C16:0, palmitic acid relative content; C16:1, palmitoleic acid relative content; C17:0, margaric acid relative content; C17:1, margaroleic acid relative content; C18:0, stearic acid relative content; C18:1, oleic acid relative content; C18:2, linoleic acid relative content; C18:3, α-linolenic acid relative content; C20:0, arachidic acid relative content; C20:1, gadoleic acid relative content; C20:2, eicosadienoic acid relative content; C22:0, behenic acid relative content; C22:1, erucic acid relative content.

## Abbreviations

SMC: seed moisture content
TFT: 2,3,5-triphenyl-2H-tetrazolium chloride
FRAP: ferric reducing antioxidant power assay
TFC: total flavonoid content
QE: quercetin equivalent
TPC: total phenolic content
GAE: gallic acid equivalent
NBT: nitroblue tetrazolium
DAB: diaminobenzidine
ROS: reactive oxygen species
MUFA: mono-unsaturated fatty acids
PUFA: polyunsaturated fatty acids
SFA: saturated fatty acids.
