# Supplementary figures and images for: Changes in nutritional quality-related traits of quinoa seeds under different storage conditions
Source: Front Nutr. 2022 Oct 17;9:995250. doi: 10.3389/fnut.2022.995250 (PMC9620721; doi:10.3389/fnut.2022.995250)

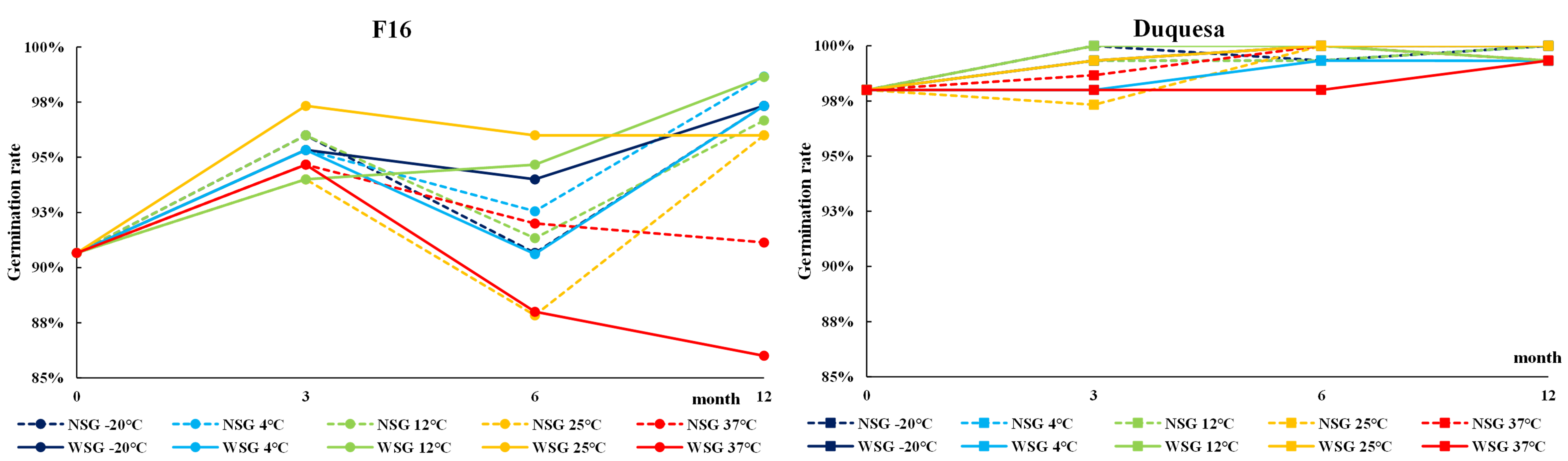

Supplement: Supplementary Figure 1 — Germination rate of quinoa 7 days after sowing throughout 12 months of storage. NSG, seeds stored without silica gel; WSG, seeds stored with silica gel. [file Image_1.tif]

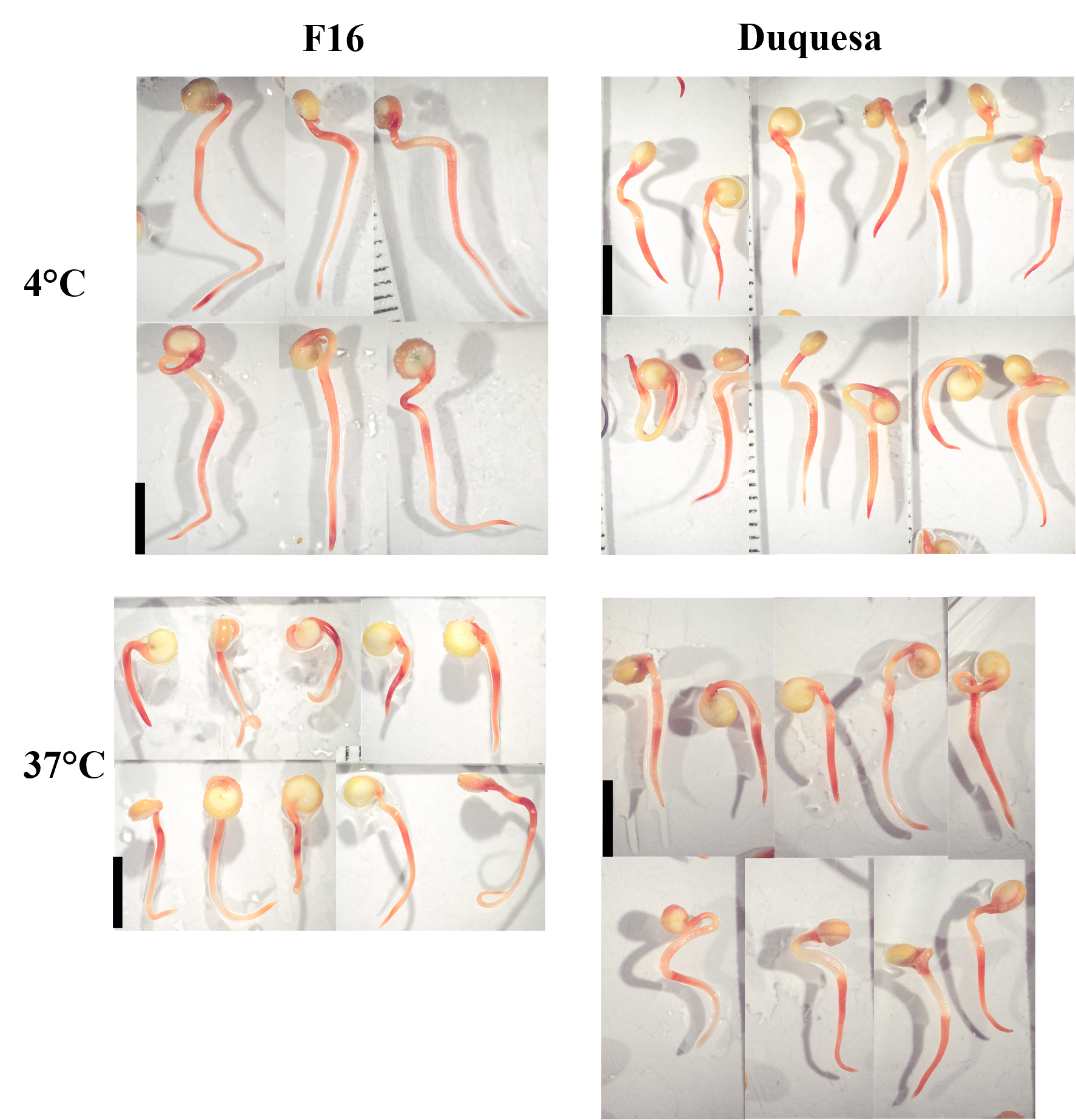

Supplement: Supplementary Figure 2 — 2,3,5-triphenyl-2H-tetrazolium chloride staining of quinoa seedlings. Seeds of F16 and Duquesa genotypes stored for 12 months at 4°C or 37°C were grown for 3 days and their sprouts were stained with TFT. Scale bars indicate 5 mm. [file Image_2.tif]

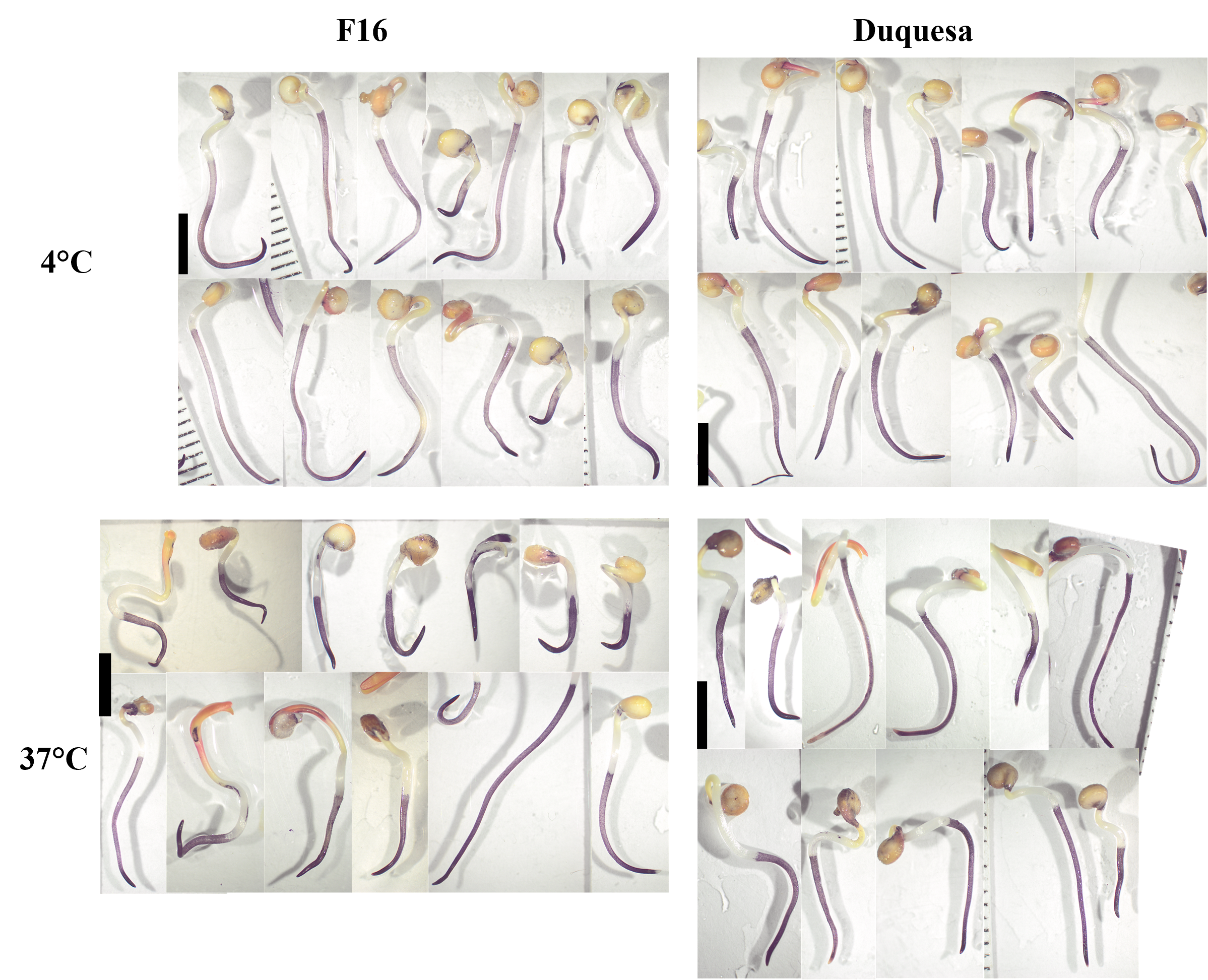

Supplement: Supplementary Figure 3 — Nitroblue tetrazolium staining of quinoa seedlings. Seeds of F16 and Duquesa genotypes stored for 12 months at 4 or 37°C were grown for 3 days and their sprouts were stained with NBT. Scale bars indicate 5 mm. [file Image_3.tif]

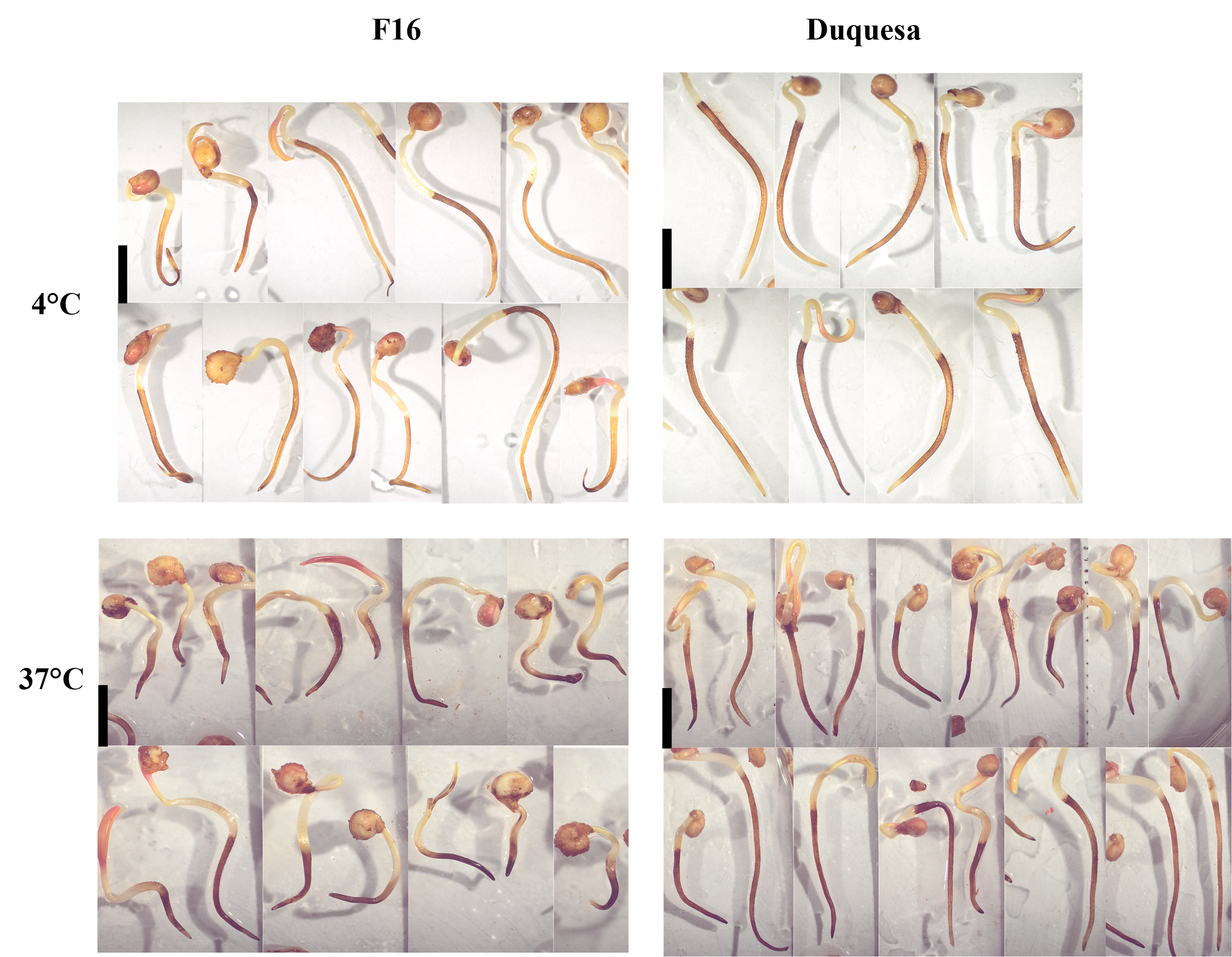

Supplement: Supplementary Figure 4 — Diaminobenzidine staining of quinoa seedlings. Seeds of F16 and Duquesa genotypes stored for 12 months at 4 or 37°C were grown for 3 days and their sprouts were stained with DAB. Scale bars indicate 5 mm. [file Image_4.tif]

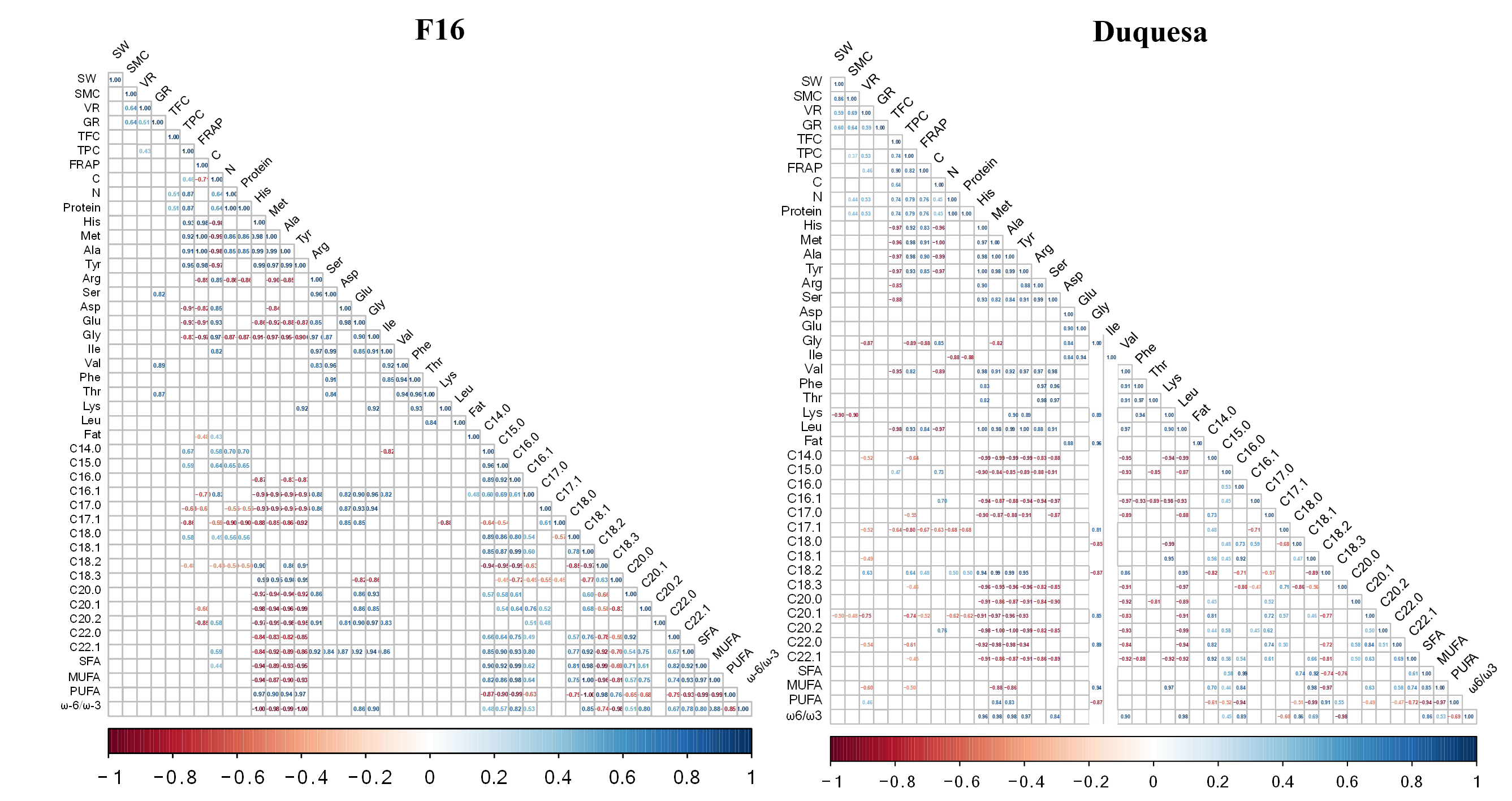

Supplement: Supplementary Figure 5 — Correlogram of variables measured in F16 and Duquesa seeds. Pearson correlation coefficients (r) are given when the correlation between variables is statistically significant (p < 0.05). Red coefficients indicate negative correlations and blue numbers show positive correlations. SW, 1000 seeds weight; SMC, seed moisture content; VR, viability rate; GR, germination 1 date after sowing; TPC, total phenolic content; FRAP, antioxidant power; C, carbon content; N, nitrogen content; C14:0, myristic acid relative content; C15:0, pentadienoic acid relative content; C16:0, palmitic acid relative content; C16:1, palmitoleic acid relative content; C17:0, margaric acid relative content; C17:1, margaroleic acid relative content; C18:0, stearic acid relative content; C18:1, oleic acid relative content; C18:2, linoleic acid relative content; C18:3, α-linolenic acid relative content; C20:0, arachidic acid relative content; C20:1, gadoleic acid relative content; C20:2, eicosadienoic acid relative content; C22:0, behenic acid relative content; C22:1, erucic acid relative content. [file Image_5.tif]
